# How to Efficiently Design 2D Materials for Electrochemical Applications Using Machine Learning

**DOI:** 10.1021/prechem.5c00046

**Published:** 2025-10-16

**Authors:** Pawin Iamprasertkun

**Affiliations:** School of Bio-Chemical Engineering and Technology, Sirindhorn International Institute of Technology, and Research Unit in Sustainable Electrochemical Intelligent, 37698 Thammasat University, Khlong Luang 12120, Pathum Thani, Thailand

## Abstract

Two dimensional (2D) materials have transitioned from lab findings to potential applications. Starting with the isolation of graphene, the field has rapidly expanded to encompass a broad spectrum of materials, including transition metal dichalcogenides, MXenes, and so on. Each of them offers unique structural, electronic, optical, and electrochemical properties. These materials have been recognized as candidates for applications in energy storage and conversion including electrocatalysts. As we approach the limits of traditional “trial-and-error” methods, the integration of statistical analysis, machine learning (ML), live (real-time) electrochemistry, and generative AI presents a compelling path forward. These tools are no longer aspirational; they are becoming essential to navigating the vast and complex design space of 2D materials for electrochemical applications in the future.

## Introduction

Over the past two decades, 2D materials have been synthesized using a variety of techniques for diverse electrochemical applications, hence, a ton of data has been generated. Therefore, ***a promising starting point for future research is the utilizing of literature data from databases***. Even simple statistical analysis in electrochemistry can provide fundamental insights into the behavior and design of 2D materials. This is because statistical tools such as regression, ANOVA,^1^ curve fitting, or Pearson/Spearman correlation serve as an essential bridge between raw data and meaningful interpretation.^2^ For example, Pearson correlation can provide an indication of which material design features enhance electrochemical activity, such as capacitance and overpotential. Performing fitting analyses can also yield insights into the dominant electrochemical mechanisms at play.^2^ More advanced methods, such as principal component analysis (PCA), can help reduce dimensionality, highlight dominant trends, and identify redundant descriptors in large data sets.^3^ This approach provides useful electrochemical insights without requiring laboratory resources and can guide newcomers toward more precise synthesis and measurement. However, data sparsity, inconsistent units, and varying test conditions limit reproducibility, so relying solely on statistical analysis may lead to misinterpretations. Yet, it remains a valuable practice for understanding data distribution and ensuring that only clean, structured, and interpretable data are fed into machine learning models, thereby building trust in downstream predictions. This topic has not been widely emphasized, as most researchers focus on experimental work; however, after COVID-19 it became central in “2D materials” and “electrochemistry,” where many materials scientists began using artificial neural networks (ANNs) to predict electrochemical features. While black-box models such as ANNs can provide strong predictive performance when hyperparameters are finely tuned, they are also prone to overfitting.^3,4^ Another major concern is the validity of the data. If incorrect data are fed into the model, the output will inevitably be flawed, no matter how precise the model is.^5^ To address this issue, employing classical machine learning approaches–such as regression or tree-based methods, and even complex structural models–may improve the accuracy of electrochemical predictions. Hence, feature-importance analysis tools such as Shapley Additive Explanations (SHAP), Partial Dependence Plots (PDP), and Feature Importance (FPI) can be applied to enable a so-called “inverted design”.^6^ ***However, it should be noted that the current approach to predicting electrochemical performance may soon reach its limits, particularly the notion of simply collecting data and applying conventional models, such as artificial neural networks (ANNs), without deeper physical insight.*** Recent research has shifted toward the use of generative AI, led by teams such as Microsoft Research, and Google DeepMind to discover materials with tailored properties for specific applications, such as energy storage and electrocatalysis.^7,8^ With established tools such as high-throughput screening,^9^ open materials databases,^10^ and machine learning models for property prediction–such as crystal graph convolutional neural networks^11^–***it is now possible to screen hundreds of thousands of materials to identify the most promising candidates followed by computational and experimental validation***. This approach greatly accelerates the discovery of improved materials, overcoming the limitations of human intuition, restricted candidate spaces, and lengthy iterative cycles, ultimately enabling the discovery of new high-performance materials for many electrochemical applications.

## Features Prediction of 2D Materials

From an electrochemical perspective, ML has been applied to predict capacitance, energy/power density, catalytic activity, and adsorption energies of 2D materials, while also reducing the computational cost of first-principles simulations.^12^ Mining experimental data and applying supervised learning methods also accelerates discovery and broadens the design space.^13^ Recent studies show that ML can predict electrocatalytic performance, provide mechanistic insights, and guide the design of efficient electrodes. Interpretability tools such as Shapley Additive Explanations (SHAP) can quantify the contribution of each design feature–e.g., surface area, doping, defects, or pore size–to electrochemical performance metrics such as capacitance and catalytic activity.^3,4^ Partial Dependence Plots (PDPs) visualize how a specific feature–such as capacitance or charge–discharge rate–affects performance while holding other features constant, helping researchers identify key synthesis (in focus) or operating parameters to optimize electrochemical behavior.^14^ “Inverse material design” can be achieved through Feature Importance (FPI) analysis, which guides experimental efforts toward the most relevant features, streamlines the material design process, and reduces reliance on trial-and-error approaches.^14^ The key challenge is translating ML “recipes” into practical synthesis. However, conducting experiments or relying on data from previous literature still limits the pace of materials discovery. By integrating computational and experimental workflows, ML could propose optimal synthesis condition including optimum surface area, doping, heterostructures, or compositions–drastically reducing the time and cost compared with traditional experimental method.

## Materials Discovery through Generative Models

Machine learning can accelerate materials discovery by predicting properties at low computational cost, but most methods still depend on experimental data or crystal structure descriptors, restricting their use to well-characterized materials. Roost (Representation Learning from Stoichiometry), developed by Goodall and Lee,^15^ overcomes this limitation by treating stoichiometric formulas as dense weighted graphs between elements and learning descriptors directly from data. This approach achieves lower errors with less data than existing structure-agnostic methods. This development has expanded into state-of-the-art graph neural networks, such as those from Google DeepMind, for materials exploration and candidate structure filtering. Using this approach, they have discovered more than a million stable structures.^8^ With generative models–such as score-based approaches–it is possible to reconstruct known or even novel stable structures using only atomic numbers as input.^16^ It is clear that developing more precise generative AI remains a major focus in industry. Microsoft Research recently introduced “*MatterGen*”, a framework trained on crystallographic data and designed for high structural validity and broad chemical space exploration. By implicitly learning symmetry and bonding rules, it offers a more general-purpose and physics-aligned approach, representing a significant improvement over earlier generative models.^7^ ***However, result validation remains a critical issue. While deep learning and automation hold great promise for accelerating materials discovery, their outputs must be carefully verified, as methodological errors can lead to overstated claims.*** For instance, incorrect or poor diffraction fits that overlook strong peaks can make it impossible to confirm the reported structures or purities.^17^ Yet, reliable validation often requires density functional theory (DFT), which can be computationally expensive.

## Autonomus Electrochemical Systems

The integration of live experimentation is beginning to transform how 2D materials are synthesized and characterized.^18^ Synthesis protocols are continuously refined based on real-time data, significantly reducing the time required to optimize material properties and ensuring high-quality material production.^19^ This approach is particularly beneficial for the synthesis of 2D materials for electrochemical applications, where minor variations in experimental conditions such as synthesis environment, solvent, exfoliation time and power, or substrate can dramatically affect the electrochemical performance. For example, in the development of WSe_2_, small changes in synthesis conditions can influence the material capacitance, catalytic activity and cycling stability.^20^ By integrating autonomous synthesis with real-time spectral analysis and ML algorithms, it is possible to rapidly map the synthesis-property relationships of materials, accelerating the optimization process and enabling the development of high-performance materials for energy storage applications.^21^ Szymanski et al. demonstrated the A-Lab (autonomous laboratory) platform for synthesizing novel materials, discovering over 41 new compounds within just 17 days by leveraging data from the Materials Project and Google DeepMind.^22^ While ML and autonomous experimentation have shown significant promise in predicting the intrinsic properties of 2D materials, a critical challenge remains in translating these properties into device-level. It is interesting to consider a closed-loop design approach, such as electrolyte design for industrial-scale electrochemical systems.^23^ However, ***there is currently a shortage of chemists and materials scientists with programming skills, as well as computer engineers with a strong foundational understanding of chemistry. As a result, communication between experts in these fields may still be limited, posing a challenge to conducting truly novel interdisciplinary research.***

## Conclusion and Outlook

A particularly underexplored yet promising direction is the integration of machine learning with “*in-situ*” or “*operando*” materials and electrochemical characterization. Most current applications still rely on static data sets derived from “*ex-situ*” measurements or DFT simulations, which fail to capture the dynamic nature of material behavior under real operating conditions. Real-time data such as changes in structural integrity, charge transfer resistance, or ion diffusion can offer valuable insights into transient processes that govern long-term performance and degradation. By embedding ML frameworks into platforms coupled with *in situ* Raman spectroscopy, electrochemical impedance spectroscopy (EIS), or synchrotron-based X-ray absorption techniques, researchers can unveil temporal correlations and early signs of failure mechanisms. This approach not only enhances predictive accuracy but also paves the way for more resilient and application tailored material designs. Looking further, the future of 2D materials synthesis will be driven by the seamless integration of machine learning, autonomous synthesis, and advanced data analytics including the use of generative AI for discover novel materials. These data centric strategies combined with high throughput experimentation, which will accelerate the rediscovery of 2D materials. Once researchers become familiar with machine learning tools (also referred to as AI), their use will resemble the way scientific calculators revolutionized the handling of complex equations. AI is poised to become an indispensable tool in materials innovation, significantly reducing development time while enhancing precision in the chemistry of 2D materials.
